# Tumor Glucose Metabolism and Its Heterogeneity on F-18 FDG PET/CT Provide Better Prognostication in Nonmetastatic Human Papillomavirus-Related Oropharyngeal Squamous Cell Carcinoma

**DOI:** 10.3390/cancers13215538

**Published:** 2021-11-04

**Authors:** Hojin Cho, Soyoung Kim, Kwanhyeong Jo, Yong Hyu Jeong, Won Jun Kang

**Affiliations:** Department of Nuclear Medicine, Severance Hospital, Yonsei University College of Medicine, Seoul 03722, Korea; hojincho@yuhs.ac (H.C.); saranghayo3@yuhs.ac (S.K.); phe_ea@naver.com (K.J.); fulif@yuhs.ac (Y.H.J.)

**Keywords:** oropharyngeal carcinoma, prognosis, FDG, PET/CT

## Abstract

**Simple Summary:**

Human papillomavirus (HPV)-related oropharyngeal squamous cell carcinoma (OPSCC) emerged as a distinct disease with a favorable prognosis, and a separate staging system was introduced. However, a subset of patients harbor a poor prognosis. We aimed to evaluate the prognostic role of metabolic parameters on baseline F-18 FDG PET/CT in patients with HPV-related OPSCC. We retrospectively reviewed patients who were diagnosed with stage I, II, and III HPV-related OPSCC using the 8th TNM staging. Metabolic features on baseline F-18 FDG PET/CT, such as higher tumor glucose metabolism derived from tumor SUV_max_ to liver SUV_mean_ ratio, and increased intratumoral heterogeneity inferred from coefficient of variation were associated with poorer progression-free survival and overall survival. Further study is warranted to address the possible implications of F-18 FDG PET/CT on treatment de-intensification in these patients.

**Abstract:**

Background: We aimed to evaluate the prognostic role of metabolic parameters on baseline F-18 fluorodeoxyglucose (FDG) PET/CT in patients with human papillomavirus (HPV)-related oropharyngeal squamous cell carcinoma (OPSCC). Methods: We retrospectively reviewed patients who were diagnosed with nonmetastatic HPV-related OPSCC using the 8th TNM staging system from 2010 to 2015 and underwent baseline F-18 FDG PET/CT. Tumor SUV_max_ to liver SUV_mean_ ratio (SUV_max_-TLR), metabolic tumor volume (MTV), tumor total lesion glycolysis to liver SUV_mean_ ratio (TLG-TLR), and coefficient of variation (CV) of the primary tumor were measured. Patients were primarily treated with surgery or radiotherapy. Endpoints were progression-free survival (PFS) and overall survival (OS). Results: Ninety consecutive patients (male, 72; female, 18) were enrolled. They were followed up for a median of 77.4 months (interquartile range, 48.4–106.4). Sixteen patients progressed, and 13 died. Multivariate analysis revealed that patients with advanced age, overall stage, and higher SUV_max_-TLR or CV had poorer PFS and OS. Conclusion: Higher SUV_max_-TLR and CV of the primary tumor on baseline F-18 FDG PET/CT were associated with poorer PFS and OS in patients with nonmetastatic HPV-related OPSCC. Further study is warranted to address the possible implications of F-18 FDG PET/CT on treatment de-intensification in these patients.

## 1. Introduction

The incidence of oropharyngeal squamous cell carcinoma (OPSCC) is increasing, with more than 90,000 new cases each year globally [1]. Human papillomavirus (HPV) infection results in an increased incidence of oropharyngeal cancer, and recent studies have shown that approximately 70% of OPSCC cases are associated with HPV in North America and Europe [2,3,4,5,6]. HPV-related OPSCC is clinically and biologically different from HPV-unrelated OPSCC [7,8,9]. HPV-related OPSCC generally occurs in a younger and healthier population with less exposure to tobacco smoke, and it has better overall survival (OS) and progression-free survival (PFS) [10,11]. The 8th edition of the American Joint Committee on Cancer staging system provides a better understanding of the clinical behavior and tumor biology, and a separate staging algorithm was introduced for HPV-related OPSCC, resembling the N classification of nasopharyngeal carcinoma [12,13]. Approximately 80% of patients with HPV-related OPSCC will probably be cured [10,14]. However, a subset of patients have a poor prognosis, and this population needs to be investigated.

Patients with early stage OPSCC may be treated with surgery or radiotherapy [15,16]. For patients with more advanced stages, concurrent cisplatin-based chemoradiotherapy is administered either definitely or as postoperative adjuvant treatment [17,18]. The current treatments are strict and cause substantial acute and late toxicity [19]. Because patients with HPV-related OPSCC have a better prognosis, a growing body of research has been focused on treatment de-intensification maintaining the current cure rates while reducing treatment-related morbidities. Although a subset of HPV-related OPSCC show a good prognosis, the early results from treatment de-intensification trials support to maintain current treatment guidelines [20,21]. Therefore, precisely identifying this good-risk population is crucial.

It is not clear that F-18 fluorodeoxyglucose (FDG) positron emission tomography/computed tomography (PET/CT) can reclassify patients with HPV-related OPSCC into different risk categories. F-18 FDG PET/CT has been widely used in staging and evaluating therapy response in patients with HPV-related OPSCC [22,23,24]. Previous studies also showed that higher maximum standardized uptake value (SUV_max_), metabolic tumor volume (MTV), and total lesion glycolysis (TLG) are associated with worse prognosis [25,26]. By contrast, some studies reported that the metabolic parameters on F-18 FDG PET/CT do not predict patients’ outcome [27,28]. These studies were based on the 7th TNM staging system, and the prognostic role of F-18 FDG PET/CT has not been validated using the 8th TNM staging system. The present study aimed to determine whether metabolic parameters on baseline F-18 FDG PET/CT improve prognostication in patients with HPV-related OPSCC using the current 8th TNM staging system.

## 2. Materials and Methods

### 2.1. Patients

We retrospectively reviewed the medical records of patients who were newly diagnosed with stage I, II, and III HPV-related OPSCC using the 8th TNM staging system and underwent F-18 FDG PET/CT scan from 2010 to 2015. The HPV status was determined using p16 immunohistochemistry. Patients with a history of other malignancies and who received any treatment prior to F-18 FDG PET/CT scans were excluded. Patients were reviewed at a multidisciplinary tumor board to determine primary treatment. In patients who received surgery as the primary treatment, postoperative radiation therapy was given to patients with pT3-4 disease, a close margin, one positive node greater than 3 cm or multiple positive nodes, lymphovascular invasion, or perineural invasion. Postoperative chemoradiotherapy was delivered to patients with extranodal extension or a positive margin.

After treatment completion, patients were followed up every 3 months for the first 2 years, then every 6 months until 5 years, and annually thereafter. Treatment response and follow-up evaluation were assessed by recording their history and performing a physical examination, neck CT, and/or MRI. F-18 FDG PET/CT, whole-body bone scan, or chest CT were obtained according to the referring physicians’ preference; follow-up imaging was performed every 3–6 months for the first 2 years and annually thereafter. Treatment response was assessed with the response evaluation criteria in solid tumors (RECIST), version 1.1.

### 2.2. F-18 FDG PET/CT

All patients underwent F-18 FDG PET/CT on either a Biograph TruePoint 40 PET/CT scanner (Siemens Healthcare, Erlangen, Germany) or a Discovery STe PET/CT scanner (GE Healthcare, Milwaukee, WI, USA). Patients fasted for at least 6 h before the scan, and peripheral blood glucose levels were no higher than 140 mg/dL before F-18 FDG injection. Approximately 5.5 MBq of F-18 FDG per kg of body weight was administered intravenously 1 h before the start of imaging. After the initial low-dose CT (Biograph TruePoint 40: 36 mA, 120 kVp; Discovery STe: 30 mA, 140 kVp), standard PET imaging was conducted from the cerebellum to the mid-thigh, with acquisition times of 2.5 min/bed position for the Biograph TruePoint 40 scanner and 3 min/bed position for the Discovery STe scanner in a three-dimensional mode. PET images were reconstructed iteratively with CT-based attenuation correction.

### 2.3. Image Analysis

All F-18 FDG PET/CT images were reviewed by two nuclear medicine physicians using MIM imaging software (MIM 6.8; MIM Software Inc., Cleveland, OH, USA). The SUV_max_ values were measured in a volume of interest (VOI) drawn on PET images. In each patient, the SUV_max_, MTV (the metabolically active volume of the tumor), TLG (the product of mean SUV and MTV), and coefficient of variation (CV; the ratio of the standard deviation to the mean) of the primary tumor were measured. Normal background liver SUV mean and standard deviation (SD) were measured by drawing a 3-cm-sized spherical VOI in the right lobe of the liver. To calculate the MTV threshold, we used the following formula: liver SUV_mean_ + (2 × liver SUV_SD_) [29]. The SUV of the VOI was calculated as follows: decay-corrected activity (kilobecquerel) per mm of tissue volume/injected F-18 FDG activity (kilobecquerel)/body weight (g). The SUV_max_ and TLG of the primary tumor were divided by mean liver SUV to mitigate variability in SUV measurements, yielding tumor SUV_max_ to liver SUV_mean_ ratio (SUV_max_-TLR), and tumor total lesion glycolysis to liver SUV_mean_ ratio (TLG-TLR), respectively.

### 2.4. Statistical Analysis

The following variables were included in univariate analyses: age, sex, ECOG performance status, stage, smoking history, primary treatment, and metabolic parameters on F-18 FDG PET/CT (SUV_max_-TLR, MTV, TLG-TLR, and CV of the primary tumor). Survival time was calculated from the date of diagnosis to the date of development of first progression or death from any cause. We used a Cox proportional-hazards model to evaluate PFS and OS. Statistical analyses were performed using R software (version 4.1.0, R Core Team, 2021, Vienna, Austria), and a *p* value < 0.05 was considered statistically significant. The optimal cutoffs for continuous variables were calculated using the maximally selected rank statistics [30].

## 3. Results

### 3.1. Patient Characteristics

**Figure 1:**
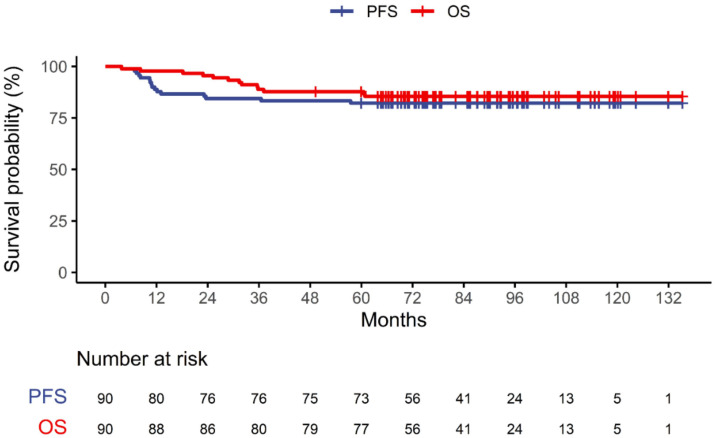
Progression-free survival (PFS) and overall survival (OS) during the follow-up period. Tick marks represent censored data.

A total of 90 consecutive patients (72 males and 18 females) were enrolled in this study. Their mean age was 57.3 (SD, 8.6) years. Among the 90 patients, 61 (67.8%), 11 (12.2%), and 18 (20.0%) had stage I, II, and III HPV-related OPSCC, respectively. Fifty-two patients (57.8%) had a smoking history equal to or less than 10 pack-years. Seventy-seven patients (85.6%) underwent surgery as primary treatment. Among them, 71 (92.2%) received adjuvant radiotherapy (*n* = 15) or chemoradiotherapy (*n* = 56). Chemotherapy was given to 76 patients (84.4%). (Table 1). The patients were followed up for a median of 77.4 months (interquartile range, 48.4 to 106.4). During the follow-up period, 16 (17.8%) had progressive disease, and 13 (14.4%) died. The two-year PFS and OS rates were 84.4% and 95.6%, respectively (Figure 1). Significant correlations were found between SUV_max_-TLR, MTV, TLG-TLR, and CV (*p* < 0.001). Therefore, only one of the metabolic parameters on F-18 FDG PET/CT was included in the multivariate analysis at a time.

### 3.2. Prognostic Values of Clinical Parameters and Metabolic Parameters of F-18 FDG PET/CT

The optimal cutoff values for age, SUV_max_-TLR, MTV, TLG-TLR, and CV in predicting PFS were 57, 4.8, 17.1, 84.2, and 27.9, respectively. Poorer PFS was associated with advanced age (≤57 (*n* = 45 (50.0%)) vs. >57 (*n* = 45 (50.0%) years); *p* = 0.04, hazard ratio (HR) = 3.21), higher overall stage (I-II vs. III; *p* < 0.001, HR = 7.21), radiotherapy as primary treatment, as compared with surgery (*p* = 0.004, HR = 4.37), SUV_max_-TLR (≤4.8 (*n* = 35 (38.9%)) vs. >4.8 (*n* = 55 (61.1%)); *p* = 0.02, HR = 11.03), MTV (≤17.1 (*n* = 75 (83.3%)) vs. >17.1 mL (*n* = 15 (16.7%)); *p* < 0.001, HR = 6.81), TLG-TLR (≤84.2 (*n* = 81 (90.0%)) vs. >84.2 (*n* = 9 (10.0%)); *p* < 0.001, HR = 8.34), and CV (≤27.9 (*n* = 43 (47.8%)) vs. >27.9% (*n* = 47 (52.2%)); *p* = 0.007, HR = 16.54). Nodal stage did not affect PFS among patients with same overall stage (all *p* values were greater than 0.05).

Two multivariate models showed significant associations between metabolic parameters and PFS. The first model (PFS model 1) included age, overall stage, primary treatment, and SUV_max_-TLR; advanced age, higher overall stage, and SUV_max_-TLR remained significant in predicting worse PFS. The second model (PFS model 4) included age, overall stage, primary treatment, and CV; advanced age, higher overall stage, and CV were significant predictors of poorer PFS (Table 2).

The optimal cutoff values for age, SUV_max_-TLR, MTV, TLG-TLR, and CV in predicting OS were 57, 4.9, 17.1, 50.0, and 27.9, respectively. Advanced age (≤57 vs. >57 years; *p* = 0.02, HR = 5.94), higher overall stage (I–II vs. III; *p* < 0.001, HR = 8.29), radiotherapy as primary treatment (*p* = 0.001, HR = 6.29), higher SUV_max_-TLR (≤4.9 (*n* = 39 (43.3%)) vs. >4.9 (*n* = 51 (56.7%)); *p* = 0.02, HR = 10.38), MTV (≤17.1 vs. >17.1 mL; *p* = 0.002, HR = 5.55), TLG-TLR (≤50.0 (*n* = 69 (76.7%)) vs. >50.0 (*n* = 21 (23.3%)); *p* = 0.001, HR = 6.48), and CV (≤27.9 vs. >27.9%; *p* = 0.01, HR = 12.64) were associated with worse OS. Nodal stage also did not affect OS among patients with the same overall stage (all *p* values were greater than 0.05).

Significant associations between metabolic parameters and OS were found in two multivariate models. The first model (OS model 1) included age, overall stage, primary treatment, and SUV_max_-TLR; advanced age, higher overall stage, and SUV_max_-TLR remained significant in predicting poorer OS. The second model (OS model 4) included age, overall stage, primary treatment, and CV; advanced age, higher overall stage, and CV were significant predictors of worse OS (Table 3).

**Figure 2:**
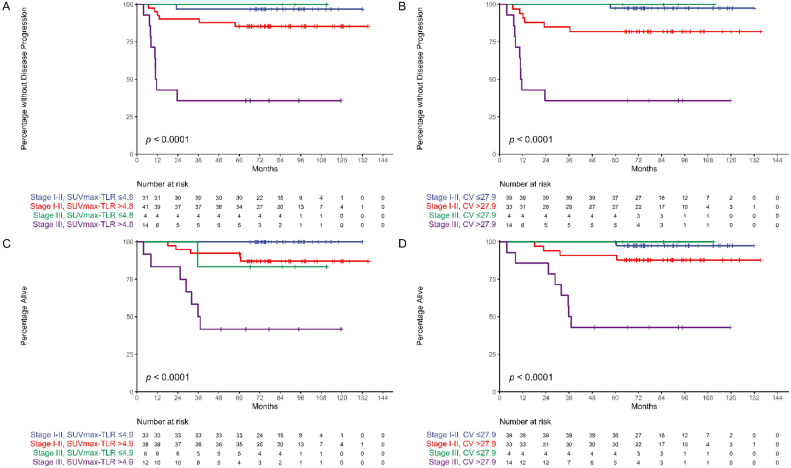
Kaplan–Meier estimates of progression-free survival (**A**,**B**) and overall survival (**C**,**D**) according to stage, and tumor SUV_max_ to liver SUV_mean_ ratio (SUV_max_-TLR) (**A**,**C**) or coefficient of variation (CV) (**B**,**D**). Global *p* values are presented to compare progression-free survival and overall survival across groups. Tick marks represent censored data.

**Figure 3:**
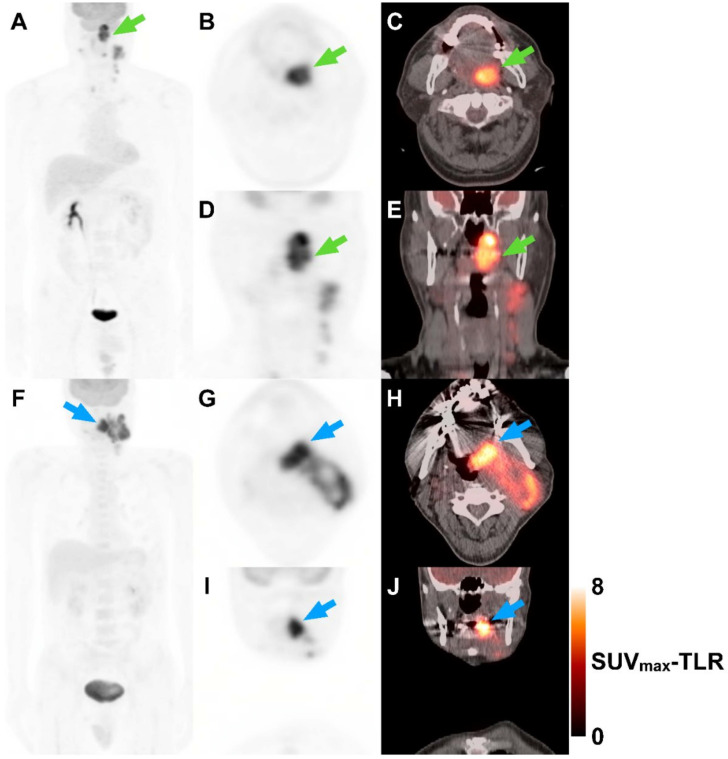
Two representative cases show that patients’ outcomes could be estimated based on metabolic parameters. A 59-year-old man with HPV-related oropharyngeal cancer squamous cell carcinoma (OPSCC) (**A**–**E**). Maximum intensity projection (**A**), axial and coronal PET (**B**,**D**), and fused PET/CT (**C**,**E**) images demonstrate left tonsillar mass with bilateral cervical lymph node metastasis. The primary tumor shows heterogeneous FDG uptake (green arrows). The TNM designation is T4N2M0 with a prognostic grouping of stage III. Tumor SUV_max_ to liver SUV_mean_ ratio (SUV_max_-TLR) and coefficient of variation (CV) are 8.0 and 34.5%, respectively. The patient progressed after 11.6 months and died after 28.8 months. Another 59-year-old man with HPV-related OPSCC (**F**–**J**). Maximum intensity projection (**F**), axial and coronal PET (**G**,**I**), and fused PET/CT (**H**,**J**) images reveal left tonsillar mass with bilateral cervical lymph node metastasis. The primary tumor shows relatively homogeneous FDG uptake (blue arrows). The TNM designation is T4N3M0 with a prognostic grouping of stage III. SUV_max_-TLR and CV are 7.0 and 27.9%, respectively. The patient has not progressed or died during the 63.8-month follow-up.

**Figure 4:**
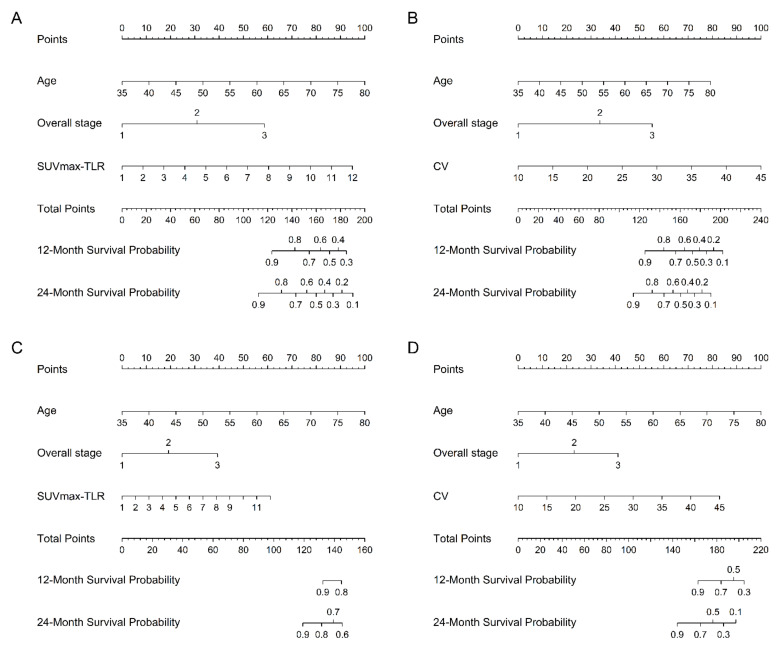
Nomograms predicting the 1- and 2-year progression-free (**A**,**B**) and overall (**C**,**D**) survival rates. To calculate predicted survival, a straight line is drawn up to the row labeled “Points” to determine the corresponding points for each factor. The total points projected on the bottom scales show the probabilities of 1- and 2-year progression-free survival or overall survival. SUV_max_-TLR, tumor SUV_max_ to liver SUV_mean_ ratio; CV, coefficient of variation.

Kaplan–Meier estimates also reveal that patients with HPV-related OPSCC can be classified into groups with different PFS and OS according to stage, and SUV_max_-TLR or CV (Figure 2). In stage I and II disease, higher SUV_max_-TLR and CV were associated with poorer OS (*p* = 0.03) and PFS (*p* = 0.02), respectively. With higher SUV_max_-TLR and CV, stage III disease showed poorer PFS and OS than stage I and II disease (*p* <0.001), respectively. Subgroup analyses in patients primarily treated with surgery also showed that metabolic parameters along with overall stage and lymphovascular invasion predicted PFS; Higher SUV_max_-TLR, MTV, TLG-TLR, or CV were associated with poorer PFS. A multivariate model with overall stage, lymphovascular invasion, and CV did not reach statistical significance to predict overall survival, but showed a trend toward shorter overall survival with higher CV. (Table 4, Table 5 and Table 6). Two representative cases with different outcomes are shown in Figure 3. Although the patients had the same stage of disease, higher SUV_max_-TLR and CV predicted poorer PFS and OS. Figure 4 demonstrates the predictive nomogram estimated for the 1- and 2-year progression-free and overall survival rates based on the selected parameters in the multivariate Cox proportional hazards models.

## 4. Discussion

Our results show that metabolic features on baseline F-18 FDG PET/CT, such as higher tumor glucose metabolism derived from SUV_max_-TLR, and increased intratumoral heterogeneity inferred from CV, were associated with poorer PFS and OS after adjusting other clinical factors in patients with nonmetastatic HPV-related OPSCC using the 8th TNM staging system. We evaluated the prognostic value of a number of metabolic parameters on baseline F-18 FDG PET/CT and compared these metabolic parameters with known prognostic indicators, such as stage and smoking history. Primary treatment and volumetric parameters such as MTV and TLG on F-18 FDG PET/CT were not significant prognostic indicators in predicting PFS and OS in multivariate analysis. In surgically treated patients, we also found that metabolic parameters were significant prognostic indicators. Currently, the TNM staging system is the most important prognostic indicator. However, even after the revision to the 8th TNM staging system, the TNM staging system does not always provide accurate prognostic prediction. This study shows the potential role of metabolic parameters on F-18 FDG PET/CT in identifying different risks in patients with HPV-related OPSCC and may aid in future de-escalation trials by revealing low-risk patients.

F-18 FDG PET/CT has been widely used in diagnosis, staging, monitoring response to therapy, and prognostication of various tumors [31,32]. Increased glycolysis and metabolic heterogeneity are associated with poorer outcomes [33,34]. A number of studies have investigated the association between SUV_max_, SUV_peak_, MTV, TLG, or intratumoral heterogeneity of primary tumor and lymph node on baseline F-18 FDG PET/CT with OS, disease-free survival (DFS), or event-free survival (EFS) in patients with HPV-related OPSCC, and the results were inconclusive. Most studies assessed the stage using the 7th TNM staging system. Higher SUV_max_, MTV, TLG, and intratumoral heterogeneity of the primary tumor or lymph node were reported as significant prognosticators of OS, DFS, or EFS. Kim et al. [25] evaluated the prognostic roles of F-18 FDG PET/CT in 86 patients with stage II–IV HPV-related OPSCC. They found that higher nodal MTV_40%_ and MTV_40%_ or TLG of combined primary tumor and node are associated with worse DFS. The patients were treated with surgery followed by radiotherapy or chemoradiotherapy. Mena et al. [26] revealed that higher SUV_max_ or MTV_50%_ with increased tumoral heterogeneity derived from a cumulative SUV-volume histogram curve is correlated with poorer EFS in 105 patients with stage I-IV HPV-related OPSCC. The treatment modalities including chemoradiotherapy and surgery, followed by chemoradiotherapy or radiotherapy, were heterogeneous in this group of patients. Floberg et al. [35] explored the significance of MTV_50%_ of the primary tumor and lymph node in 153 patients with stage I-III HPV-related OPSCC using the 8th TNM staging system. These patients were treated with radiotherapy or surgery, followed by radiotherapy combined with/without chemotherapy. Higher MTV is associated with worse OS, EFS, and distant metastasis-free survival. However, some studies reported no significant association between metabolic parameters, such as SUV_max_, SUV_peak_, MTV_33%_, or MTV_41%_, and TLG of the primary tumor in predicting OS, DFS, or local control in patients with stage I-IV HPV-related OPSCC [27,28]. These studies also included patients with HPV-unrelated OPSCC or other head and neck cancers, and the patients were treated with chemoradiotherapy or radiotherapy.

The heterogeneous results may be attributed to the differences in the patient population in terms of prognosis, primary treatment, and quantification of metabolic parameters. As stated earlier, HPV-related OPSCC emerged as a distinct disease with a favorable prognosis. Current treatment is intended for tobacco-related head and neck cancers and it may be more intensive than it needed to be cured. In this regard, various approaches have been attempted to de-escalate current treatment while maintaining the current cure rate. An earlier study revealed that patients with N2c HPV-related OPSCC using the 6th TNM staging system show impaired distant control, suggesting a subset of patients with HPV-related OPSCC carry a poorer prognosis [36]. T4 or N3 disease and tobacco exposure for more than 10 pack-years are known to be associated with higher risk. Two randomized trials have assessed radiotherapy with cisplatin or cetuximab, a less toxic alternative to cisplatin, in patients with HPV-related OPSCC and found a significant benefit in OS and PFS in favor of cisplatin. In the subgroup analysis which only includes low-risk patients (T1-T3, N0-N2 (using the 7th TNM staging system), and non-smokers), the survival benefit in favor of cisplatin was still observed [37,38]. The above findings prompt the need for well-defined, low-risk patients for future de-escalation trials.

SUV has been widely used to quantify PET images and regarded as a robust and reproducible parameter for analyzing patients. However, various sources of bias can affect SUV measurement. Patient weight, blood glucose level, administered activity, the time elapsed from the injection, and imaging protocol may have contributed to the bias [39,40,41]. MTV and TLG have also received considerable attention to quantify various tumors, and are assumed to be stronger predictors than SUV_max_ in head and neck cancer [42]. However, no consensus has been reached regarding threshold methods and their prognostic significance [43,44]. To mitigate these methodological differences, we adopted the tumor to liver ratio for SUV_max_ and TLG and set the threshold for MTV based on liver uptake [29,45].

Efforts have been made to improve risk stratification in patients with HPV-related OPSCC. Most studies have focused on patients managed with definitive chemoradiotherapy and showed that prognostication can be improved by incorporating advanced T stage and N stage based on nasopharyngeal cancer N categories [10,12,36,46,47]. A study explored surgically treated patients and revealed that a composite risk stratification system which includes pathologic adverse features such as lymphovascular invasion, surgical margins, extranodal extension, advanced T stage, and the number of metastatic lymph nodes improve prognostication [48]. However, these models need further validation. Further refinement of prognostication for patients with HPV-OPSCC will provide appropriate treatment, and incorporation of metabolic parameters could be beneficial for improvement of prognostication.

This study has some limitations. The design is retrospective, and a small number of patients were analyzed. Moreover, primary treatment was varied among the patients, and most patients were primarily treated with surgery. Thus, inherent biases may have affected the results. Further validation is warranted in a large number of patients with a prospective design to assess potential prognostic roles of metabolic parameters.

## 5. Conclusions

We showed that higher tumor glucose uptake and its heterogeneity on F-18 FDG PET/CT were associated with poorer PFS and OS in patients with nonmetastatic HPV-related OPSCC. These findings imply that the metabolic parameters on F-18 FDG PET/CT may aid in better stratification along with the current 8th TNM staging system in patients with HPV-related OPSCC and in designing future de-escalation trials.

## Figures and Tables

**Table 1:** Patient characteristics.

Characteristic	All Patients (*n* = 90)
Age, mean ± SD, years	57.3 ± 8.6
Sex, *n* (%)	
Male	72 (80.0)
Female	18 (20.0)
ECOG, *n* (%)	
0	33 (36.7)
1	57 (63.3)
Overall stage, *n* (%)	
Stage I	61 (67.8)
Stage II	11 (12.2)
Stage III	18 (20.0)
Tumor stage, *n* (%)	
T1	19 (21.1)
T2	45 (50.0)
T3	8 (8.9)
T4	18 (20.0)
Nodal stage, *n* (%)	
N0	9 (10.0)
N1	68 (75.6)
N2	12 (13.3)
N3	1 (1.1)
Smoking history, *n* (%)	
Never or ≤10 pack-years	52 (57.8)
>10 pack-years	38 (42.2)
Primary treatment, *n* (%)	
Surgery	77 (85.6)
Surgical approach, *n* (%)	
Open	37 (48.1)
Robotic	40 (51.9)
Resection margin, *n* (%)	
Negative	52 (67.5)
Positive	25 (32.5)
Extracapsular spread, *n* (%)	
Negative	33 (42.9)
Positive	44 (57.1)
Lymphovascular invasion, *n* (%)	
Negative	52 (67.5)
Positive	25 (32.5)
Perineural invasion, *n* (%)	
Negative	66 (85.7)
Positive	11 (14.3)
Adjuvant therapy, *n* (%)	
None	6 (7.8)
Radiotherapy	15 (19.5)
Chemoradiotherapy	56 (72.7)
Radiotherapy	13 (14.4)
Chemotherapy, *n* (%)	
Yes	76 (84.4)
No	14 (15.6)
SUV_max_-TLR, median (range)	5.4 (1.5–11.0)
MTV, median (range), mL	7.9 (0.3–61.5)
TLG-TLR, median (range)	26.4 (1.3–243.7)
CV, mean ± SD, %	27.8 ± 6.3

SUV_max_-TLR, tumor SUV_max_ to liver SUV_mean_ ratio; MTV, metabolic tumor volume; TLG-TLR, tumor total lesion glycolysis to liver SUV_mean_ ratio; CV, coefficient of variation.

**Table 2:** Univariate and multivariate analyses for progression-free survival.

Characteristic	Hazard Ratio (95% CI)	*p* *
Univariate Analysis		
Age		0.04
≤57 years		
>57 years	3.21 (1.03–9.95)	
Sex		0.44
Male		
Female	0.56 (0.13–2.45)	
ECOG		0.13
0		
1	2.65 (0.75–9.30)	
Smoking history		0.24
Never or ≤10 pack-years		
>10 pack-years	1.80 (0.67–4.84)	
Overall stage		<0.001
I–II		
III	7.21 (2.67–19.45)	
Primary treatment		0.004
Surgery		
Radiotherapy	4.37 (1.58–12.07)	
Chemotherapy		0.73
Yes	1.30 (0.29–5.70)	
No		
SUV_max_-TLR		0.02
≤4.8		
>4.8	11.03 (1.46–83.53)	
MTV		<0.001
≤17.1 mL		
>17.1 mL	6.81 (2.54–18.27)	
TLG-TLR		<0.001
≤84.2		
>84.2	8.34 (2.97–23.45)	
CV		0.007
≤27.9%		
>27.9%	16.54 (2.18–125.3)	
Multivariate analysis including age, overall stage, SUV_max_-TLR, and primary treatment (PFS model 1)		
Age (≤57 vs. >57 years)	4.08 (1.29–12.93)	0.02
Overall stage (I–II vs. III)	5.36 (1.69–17.03)	0.004
Primary treatment (surgery vs. radiotherapy)	1.78 (0.53–5.97)	0.35
SUV_max_-TLR (≤4.8 vs. >4.8)	9.92 (1.26–77.86)	0.03
Multivariate analysis including age, overall stage, MTV, and primary treatment (PFS model 2)		
Age (≤57 vs. >57 years)	3.67 (1.16–11.55)	0.03
Overall stage (I–II vs. III)	3.73 (1.15–12.04)	0.03
Primary treatment (surgery vs. radiotherapy)	1.58 (0.43–5.88)	0.49
MTV (≤17.1 vs. >17.1 mL)	3.30 (0.90–12.04)	0.07
Multivariate analysis including age, overall stage, TLG-TLR, and primary treatment (PFS model 3)		
Age (≤57 vs. >57 years)	4.66 (1.39–15.62)	0.01
Overall stage (I–II vs. III)	3.63 (1.13–11.64)	0.03
Primary treatment (surgery vs. radiotherapy)	1.92 (0.56–6.63)	0.30
TLG-TLR (≤84.2 vs. >84.2)	3.80 (0.94–15.30)	0.06
Multivariate analysis including age, overall stage, CV, and primary treatment (PFS model 4)		
Age (≤57 vs. >57 years)	5.13 (1.55–16.95)	0.007
Overall stage (I–II vs. III)	6.62 (1.86–23.63)	0.004
Primary treatment (surgery vs. radiotherapy)	1.29 (0.36–4.60)	0.70
CV (≤27.9 vs. >27.9%)	16.77 (2.05–136.96)	0.009

* Wald test. CI, confidence interval; SUV_max_-TLR, tumor SUV_max_ to liver SUV_mean_ ratio; MTV, metabolic tumor volume; TLG-TLR, tumor total lesion glycolysis to liver SUV_mean_ ratio; CV, coefficient of variation; PFS, progression-free survival.

**Table 3:** Univariate and multivariate analyses for overall survival.

Characteristic	Hazard Ratio (95% CI)	*p* *
Univariate Analysis		
Age		0.02
≤57 years		
>57 years	5.94 (1.32–26.80)	
Sex		0.66
Male		
Female	0.71 (0.16–3.20)	
ECOG		0.12
0		
1	3.32 (0.74–14.99)	
Smoking history		0.36
Never or ≤10 pack-years		
>10 pack-years	1.66 (0.56–4.94)	
Overall stage		<0.001
I–II		
III	8.29 (2.70–25.47)	
Primary treatment		0.001
Surgery		
Radiotherapy	6.29 (2.10–18.79)	
Chemotherapy		0.95
Yes	0.95 (0.21–4.31)	
No		
SUV_max_-TLR		0.02
≤4.9		
>4.9	10.38 (1.35–79.89)	
MTV		0.002
≤17.1 mL		
>17.1 mL	5.55 (1.86–16.59)	
TLG-TLR		0.001
≤50.0		
>50.0	6.48 (2.12–19.86)	
CV		0.01
≤27.9%		
>27.9%	12.64 (1.64–97.24)	
Multivariate analysis including age, overall stage, SUV_max_-TLR, and primary treatment (OS model 1)		
Age (≤57 vs. >57 years)	8.24 (1.75–38.85)	0.008
Overall stage (I–II vs. III)	4.57 (1.20–17.38)	0.03
Primary treatment (surgery vs. radiotherapy)	3.23 (0.85–12.27)	0.08
SUV_max_-TLR (≤4.9 vs. >4.9)	10.16 (1.29–79.70)	0.03
Multivariate analysis including age, overall stage, MTV, and primary treatment (OS model 2)		
Age (≤57 vs. >57 years)	7.55 (1.59–35.88)	0.01
Overall stage (I–II vs. III)	3.99 (1.08–14.79)	0.04
Primary treatment (surgery vs. radiotherapy)	2.83 (0.68–11.76)	0.15
MTV (≤17.1 vs. >17.1 mL)	2.74 (0.67–11.13)	0.16
Multivariate analysis including age, overall stage, TLG-TLR, and primary treatment (OS model 3)		
Age (≤57 vs. >57 years)	7.32 (1.56–34.28)	0.01
Overall stage (I–II vs. III)	3.51 (0.94–13.11)	0.06
Primary treatment (surgery vs. radiotherapy)	3.29 (0.88–12.31)	0.08
TLG-TLR (≤50.0 vs. >50.0)	3.28 (0.94–11.42)	0.06
Multivariate analysis including age, overall stage, CV, and primary treatment (OS model 4)		
Age (≤57 vs. >57 years)	8.17 (1.73–38.54)	0.008
Overall stage (I–II vs. III)	5.42 (1.25–23.53)	0.02
Primary treatment (surgery vs. radiotherapy)	2.11 (0.50–8.85)	0.31
CV (≤27.9 vs. >27.9%)	10.64 (1.27–89.43)	0.03

* Wald test. CI, confidence interval; SUV_max_-TLR, tumor SUV_max_ to liver SUV_mean_ ratio; MTV, metabolic tumor volume; TLG-TLR, tumor total lesion glycolysis to liver SUV_mean_ ratio; CV, coefficient of variation; OS, overall survival.

**Table 4:** Patient characteristics for patients who received surgery as the primary treatment.

Characteristic	Surgically Treated Patients (*n* = 77)
Age, mean ± SD, years	57.4 ± 8.4
Sex, *n* (%)	
Male	64 (83.1)
Female	13 (16.9)
ECOG, *n* (%)	
0	30 (39.0)
1	47 (61.0)
Smoking history, *n* (%)	
Never or ≤10 pack-years	42 (54.5)
>10 pack-years	35 (45.5)
Overall stage, *n* (%)	
Stage I	58 (75.3)
Stage II	8 (10.4)
Stage III	11 (14.3)
Tumor stage, *n* (%)	
T1	16 (20.8)
T2	43 (55.8)
T3	7 (9.1)
T4	11 (14.3)
Nodal stage, *n* (%)	
N0	7 (9.1)
N1	64 (83.1)
N2	5 (6.5)
N3	1 (1.3)
Chemotherapy, *n* (%)	
Yes	63 (81.8)
No	14 (18.2)
SUV_max_-TLR, median (range)	5.3 (1.5–11.0)
MTV, median (range), mL	7.0 (0.3–46.1)
TLG-TLR, median (range)	23.1 (1.3–243.7)
CV, mean ± SD, %	27.5 ± 6.3

SUV_max_-TLR, tumor SUV_max_ to liver SUV_mean_ ratio; MTV, metabolic tumor volume; TLG-TLR, tumor total lesion glycolysis to liver SUV_mean_ ratio; CV, coefficient of variation.

**Table 5:** Univariate and multivariate analyses for progression-free survival for patients who received surgery as the primary treatment.

Characteristic	Hazard Ratio (95% CI)	*p* *
Univariate Analysis		
Age		0.22
≤57 years		
>57 years	2.32 (0.60–8.96)	
Sex		NR
Male		
Female	NR	
ECOG		0.23
0		
1	2.61 (0.55–12.29)	
Smoking history		0.36
Never or ≤10 pack-years		
>10 pack-years	1.82 (0.51–6.44)	
Overall stage		0.012
I–II		
III	5.08 (1.43–18.05)	
Surgical approach		0.41
Open		
Robotic	0.59 (0.17–2.09)	
Resection margin		0.21
Negative		
Positive	2.23 (0.64–7.69)	
Extracapsular spread		0.15
Negative		
Positive	3.10 (0.66–14.58)	
Lymphovascular invasion		0.01
Negative		
Positive	5.57 (1.44–21.55)	
Perineural invasion		0.14
Negative		
Positive	2.75 (0.71–10.63)	
Adjuvant therapy		
None		
Radiotherapy	1.16 (0.12–11.19)	0.90
Chemoradiotherapy	0.61 (0.07–5.05)	0.65
Chemotherapy		0.86
Yes	0.87 (0.18–4.08)	
No		
SUV_max_-TLR		0.003
≤7.1		
>7.1	7.05 (1.97–25.17)	
MTV		0.003
≤17.1 mL		
>17.1 mL	6.80 (1.91–24.26)	
TLG-TLR		0.003
≤70.3		
>70.3	7.05 (1.97–25.17)	
CV		0.007
≤29.8%		
>29.8%	8.31 (1.76–39.20)	
Multivariate analysis including overall stage, lymphovascular invasion, and SUV_max_-TLR (PFS model 1)		
Overall stage (I–II vs. III)	4.74 (1.20–18.68)	0.03
Lymphovascular invasion (negative vs. positive)	6.30 (1.58–25.18)	0.009
SUV_max_-TLR (≤7.1 vs. >7.1)	4.43 (1.16–16.89)	0.03
Multivariate analysis including overall stage, lymphovascular invasion, and MTV (PFS model 2)		
Overall stage (I–II vs. III)	4.05 (0.98–16.75)	0.054
Lymphovascular invasion (negative vs. positive)	6.87 (1.73–27.28)	0.006
MTV (≤17.1 vs. >17.1 mL)	5.29 (1.31–21.45)	0.02
Multivariate analysis including overall stage, lymphovascular invasion, and TLG-TLR (PFS model 3)		
Overall stage (I–II vs. III)	4.74 (1.20–18.68)	0.03
Lymphovascular invasion (negative vs. positive)	6.30 (1.58–25.18)	0.009
TLG-TLR (≤70.3 vs. >70.3)	4.43 (1.16–16.89)	0.03
Multivariate analysis including overall stage, lymphovascular invasion, and CV (PFS model 4)		
Overall stage (I–II vs. III)	6.74 (1.69–26.88)	0.007
Lymphovascular invasion (negative vs. positive)	5.68 (1.37–23.59)	0.02
CV (≤29.8 vs. >29.8%)	6.40 (1.33–30.79)	0.02

Hazard ratio is not reported for sex because of the low numbers of events. * Wald test. CI, confidence interval; NR, not reported; SUV_max_-TLR, tumor SUV_max_ to liver SUV_mean_ ratio; MTV, metabolic tumor volume; TLG-TLR, tumor total lesion glycolysis to liver SUV_mean_ ratio; CV, coefficient of variation; PFS, progression-free survival.

**Table 6:** Univariate and multivariate analyses for overall survival for patients who received surgery as the primary treatment.

Characteristic	Hazard Ratio (95% CI)	*p* *
Univariate Analysis		
Age		0.10
≤57 years		
>57 years	6.05 (0.73–50.25)	
Sex		NR
Male		
Female	NR	
ECOG		0.21
0		
1	3.89 (0.47–32.31)	
Smoking history		0.55
Never or ≤10 pack-years		
>10 pack-years	1.58 (0.35–7.07)	
Overall stage		0.03
I–II		
III	5.34 (1.19–23.94)	
Surgical approach		0.22
Open		
Robotic	0.36 (0.07–1.83)	
Resection margin		0.16
Negative		
Positive	2.95 (0.66–13.20)	
Extracapsular spread		0.15
Negative		
Positive	4.68 (0.56–38.85)	
Lymphovascular invasion		0.04
Negative		
Positive	5.76 (1.12–29.70)	
Perineural invasion		0.28
Negative		
Positive	2.49 (0.48–12.86)	
Adjuvant therapy		0.15
Radiotherapy		
Chemoradiotherapy	0.33 (0.07–1.49)	
Chemotherapy		0.42
Yes	0.51 (0.10–2.62)	
No		
SUV_max_-TLR		0.07
≤5.3		
>5.3	7.33 (0.88–60.90)	
MTV		0.07
≤15.9 mL		
>15.9 mL	4.08 (0.91–18.26)	
TLG-TLR		0.24
≤9.1		
>9.1	3.61 (0.43–30.00)	
CV		0.02
≤29.8%		
>29.8%	11.79 (1.42–98.02)	
Multivariate analysis including overall stage, lymphovascular invasion, and CV		
Overall stage (I–II vs. III)	4.93 (1.02–23.83)	0.047
Lymphovascular invasion (negative vs. positive)	4.55 (0.85–24.33)	0.08
CV (≤29.8 vs. >29.8%)	8.18 (0.96–69.95)	0.06

Hazard ratio are not reported for sex and no adjuvant therapy because of the low numbers of events. * Wald test. CI, confidence interval; NR, not reported; SUV_max_-TLR, tumor SUV_max_ to liver SUV_mean_ ratio; MTV, metabolic tumor volume; TLG-TLR, tumor total lesion glycolysis to liver SUV_mean_ ratio; CV, coefficient of variation; OS, overall survival.

## Data Availability

The data analyzed in the current study are available from the corresponding author on reasonable request.
